# Associations of neutrophil-to-lymphocyte ratio with intracranial and extracranial atherosclerotic stenosis

**DOI:** 10.3389/fneur.2022.966022

**Published:** 2022-09-20

**Authors:** Yu Xie, Zhenxing Liu, Bitang Dan, Li Zou, Lei Zhang, Renwei Zhang, Huagang Li, Qi Cai, Nadire Aiziretiaili, Shanling Ren, Yumin Liu

**Affiliations:** ^1^Department of Neurology, Zhongnan Hospital of Wuhan University, Wuhan, China; ^2^Department of Neurology, The First People's Hospital of Kashi Prefecture, Kashi, China

**Keywords:** neutrophil-to-lymphocyte ratio, atherosclerosis, intracranial stenosis, extracranial stenosis, digital subtraction angiography

## Abstract

**Background:**

Neutrophil-to-lymphocyte ratio (NLR) has been shown to be an important inflammatory maker. This study aims to investigate the association of NLR with intracranial and extracranial atherosclerotic stenosis.

**Methods:**

We retrospectively recruited patients who underwent digital subtraction angiography (DSA) for evaluating intracranial/extracranial stenosis in the Zhongnan Hospital of Wuhan University from January 2017 to October 2021. Clinical characteristics, DSA data, blood routine, and lipid profile were recorded. Logistic regression was used to evaluate the association of NLR and intercranial/extracranial atherosclerotic stenosis in three aspects: distribution of stenosis, whether the stenosis is symptomatic, and degree of stenosis.

**Results:**

A total of 1,129 patients were included in our analysis, with a median age of 62 y (interquartile range 55–68), and a median admission NLR of 2.39 (interquartile range 1.84–3.42). A total of 986 patients presented intracranial and/or extracranial atherosclerotic stenosis. Increased NLR were associated with intracranial stenosis [odds ratio (OR), 1.54; 95% CI, 1.27–1.85; *p* < 0.001], extracranial stenosis (OR, 1.56; 95% CI, 1.25–1.96; *p* < 0.001), and combined intracranial/extracranial stenosis (OR, 1.61; 95% CI, 1.28–2.03; *p* < 0.001). After adjustment of potential factors, higher NLR were independently associated with symptomatic stenosis (OR, 1.16; 95% CI, 1.05–1.27; *p* = 0.003) and degree of stenosis (OR, 1.32; 95% CI, 1.17–1.49; *p* < 0.001). Compared with the first quartile NLR, the second, third, and fourth quartiles NLR were independent risk factors for symptomatic stenosis and stenosis degree (both *p* for trend <0.001).

**Conclusion:**

Increased NLR is an important factor associated with both intracranial and extracranial atherosclerotic stenosis. Patients with symptomatic intracranial/extracranial atherosclerotic stenosis or a more severe degree of stenosis presented elevated NLR levels.

## Introduction

The cerebrovascular disease remains a leading cause of death and disability globally (1). Atherosclerosis, a chronic pathology ranging from vessel wall thickening to hemodynamically luminal stenosis, is the most common cause of cerebrovascular disease (2). Traditionally, atherosclerosis was considered as a cholesterol storage disease by reservation of lipoproteins, such as low-density lipoprotein cholesterol. Increasing evidence has suggested that atherosclerosis is a chronic inflammatory process, with the accumulation of lipids and inflammatory cells, such as macrophages, neutrophils, and lymphocytes (3). In the progression of atherosclerosis, modification of lipoproteins activates the infiltration of inflammatory cells in the intima, which responds by secreting pro-inflammatory chemokines and cytokines, promoting further recruitment of inflammatory cells of myeloid origin (4).

The neutrophil-to-lymphocyte ratio (NLR) is an indicator of inflammatory status and reflects the relationship between innate and adaptive cellular immune response during various pathological processes (5). Neutrophil-to-lymphocyte ratio has been suggested as a prognostic factor in several disorders, including ischemic stroke. Admission NLR was reported to be independently associated with stroke severity and could predict the severity and short-term outcome in stroke patients (6). Another study concluded that high NLR was associated with hemorrhagic transformation and 3-month mortality in ischemic stroke patients (7). In patients with stroke, after endovascular thrombectomy, NLR has been presented as a biomarker of intracranial hemorrhage (8). In a recent study, NLR was reported to play an important role in post-thrombolysis early neurological deterioration (9). A meta-analysis demonstrated that elevated NLR was significantly associated with poor prognosis of both ischemic and hemorrhagic stroke patients. Patients with stroke with high NLR presented a 1.1 to 1.3-fold of increased risk of poor outcomes (10). Neutrophil-to-lymphocyte ratio was also reported to be associated with carotid artery stenosis. In a retrospective study, NLR was reported to be correlated with carotid artery stenosis in male patients with ischemic stroke (11). In a study, NLR was suggested as a meaningful biomarker for the prediction of carotid plaque vulnerability and occurrence of vulnerable carotid plaque in stroke patients (12). However, few studies have explored the association between NLR and the severity and distribution of cerebrovascular stenosis (13, 14). It is worth noting that few of these studies have evaluated arterial stenosis by digital subtraction angiography (DSA), which is the standard for diagnosing intracranial and extracranial stenosis. Besides NLR, monocyte to high-density lipoprotein (MHR) was also an inflammatory marker and was reported to be associated with the degree and distribution of cerebrovascular stenosis (15).

The present study aimed to investigate the relationship between NLR and intracranial/extracranial atherosclerotic stenosis, which was evaluated by DSA. The relationship between them was analyzed from the aspects of stenosis distribution, symptom, and severity, in order to provide new insights into the pathological process of cerebral artery stenosis, and guide clinical treatment.

## Subjects and methods

### Patient selection

We analyzed consecutive patients who underwent cerebrovascular DSA examination in the department of neurology at the Zhongnan Hospital of Wuhan University from January 2017 to October 2021. The exclusion criteria were as follows: non-Chinese nationality; younger than 45-y-old; incomplete DSA data or laboratory tests; evidence of cardiogenic embolism, such as the history of atrial fibrillation; artery stenosis caused by dissection; hemorrhagic stroke; subarachnoid hemorrhage; moyamoya disease; fibromuscular dysplasia; arteriovenous malformation; aneurysm; signs of acute infection; tumor; hematological system disorder; severe liver, and kidney function impairment. This study was approved by the Clinical Research Ethics Committee of Zhongnan Hospital of Wuhan University (Ref. No: 2022106K).

### Data collection and analysis

Basic clinical data were collected from the hospital information manage system, namely gender, age, previous medical history (hypertension, diabetes mellitus, ischemic stroke), previous/current smoking, laboratory measurements within 24 h after admission, including lipid profile and blood routine (WBC, platelet, neutrophil, lymphocyte, and monocyte). The NLR was calculated by dividing the neutrophil by the lymphocyte. The MHR was calculated by dividing the monocyte by the high-density lipoprotein (HDL).

Intracranial arteries included C6-C7 segments of the internal carotid artery (ICA), M1-M2 segments of the middle cerebral artery (MCA), A1-A2 segment of the anterior cerebral artery (ACA), P1-P2 segment of the posterior cerebral artery (PCA), V4 segment of vertebral artery (VA), and basilar artery (BA). Subclavian artery, V1–V3 segments of the vertebral artery, common carotid artery, and C1–C5 segments of the internal carotid artery (ICA) are classified as extracranial arteries. The degree of stenosis was assessed according to the Warfarin-Aspirin Symptomatic Intracranial Disease Study (16), which was calculated as follows: degree of stenosis (%) = (1-diameter at the narrowest point of the narrow segment/the diameter of the proximal normal vessel) × 100%. According to the degree of stenosis, the patients were divided into a mild stenosis group (stenosis degree of 49% or less), a moderate stenosis group (stenosis degree of 50–69%), and a severe stenosis group (stenosis degree of 70–99% or occlusion). Patients were assigned to a symptomatic group in case of transient ischemic attack (TIA) and/or ischemic stroke in the territory of the stenotic artery within the proceeding 1 month, according to the definition of SAMMPRIS and VISSIT studies (17, 18). Diagnosis of the TIA and ischemic stroke were according to the AHA/ASA definition (19). According to the definitions, patients were grouped in three different ways: non-stenosis/intracranial stenosis/extracranial stenosis/combined intracranial and extracranial stenosis; asymptomatic stenosis/symptomatic stenosis; and mild/moderate/severe stenosis. The DSA data were assessed independently by two neurointerventionists who have more than 5 y of experience in interpreting DSA images; a third neurointerventiaonist confirmed the results when disagreements occurred.

### Statistical analysis

Baseline characteristics were compared between patients in different groups, namely, sex, age, previous history, blood routine test, and lipid profile. Continuous variables are presented as mean with SD for Gaussian distribution and median with interquartile range (IQR) for non-Gaussian distribution; categorical variables are presented as proportions. Comparisons were performed by the Kruskal–Wallis test and chi-square test, respectively. The *p*-values were two-sided and *p* < 0.05 was chosen as the significance level. Univariate and multivariable logistic regression were applied to evaluate the association between factors (including NLR and MHR) and arterial stenosis status. An unadjusted odds ratio (OR) with a 95% CI was estimated by univariate regression. Variables with *p* < 0.05 in the univariate analysis were included in the multivariate models. To evaluate the capacity of NLR to predict the stenosis distribution, receiver operating characteristic (ROC) curves were performed and the area under the curve (AUC) was calculated. All the statistical analyses were performed using statistical software R (version 4.0.5) (20).

## Results

**Figure 1:**
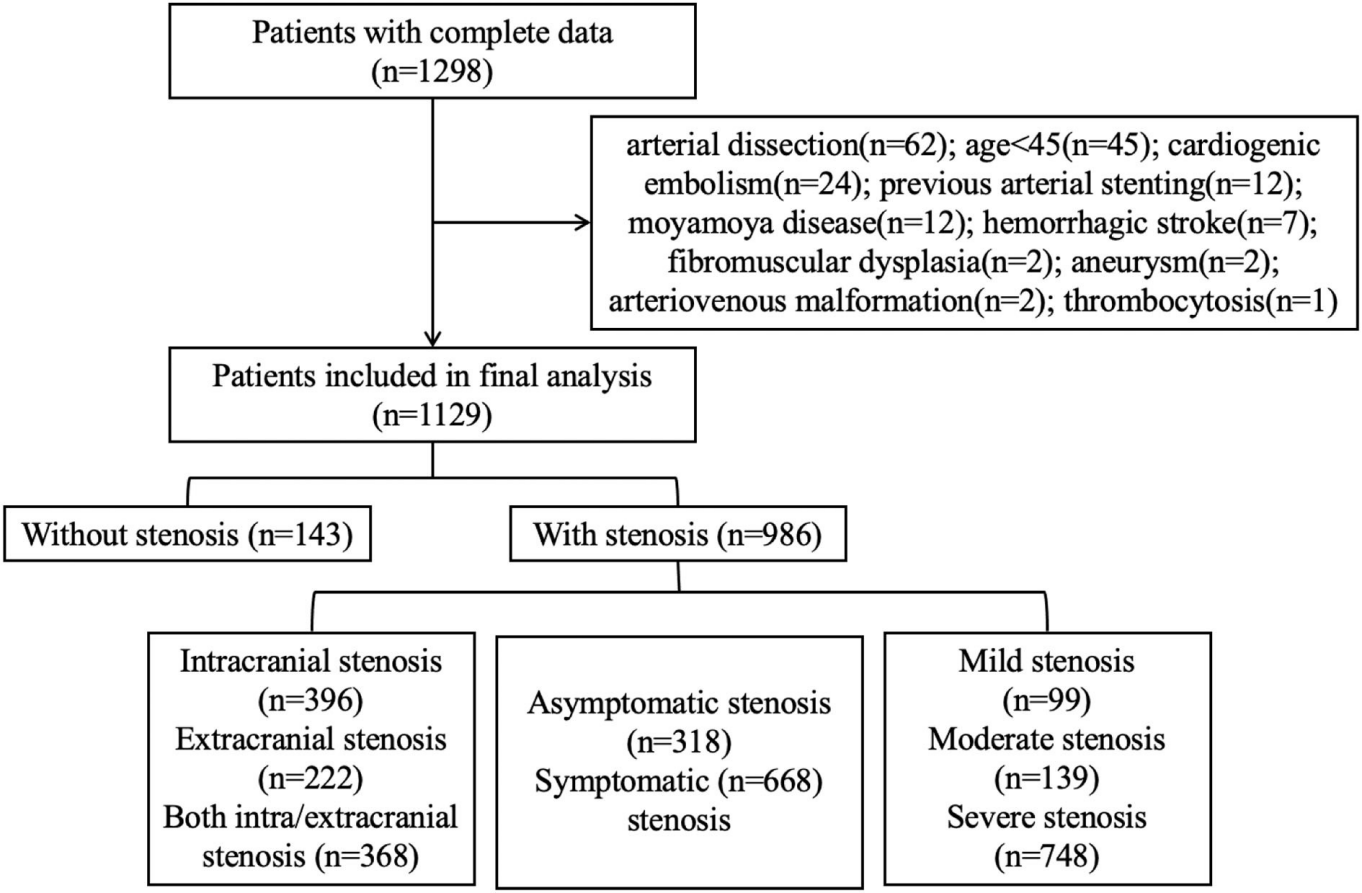
Flowchart.

Of the 1,298 patients with complete DSA and laboratory data, 1,129 patients were finally recruited for our analysis (Figure 1). The cohort was composed of 805 (71.3%) men and had a median age of 62 (IQR 55–68) y. The median admission NLR for all patients was 2.39 (IQR 1.84–3.42). Among all the patients, 143 had no intracranial or extracranial stenosis, 396 had only intracranial stenosis, 222 had only extracranial stenosis, and 368 had both stenosis; 668 had symptomatic stenosis while 318 had asymptomatic stenosis. The patients with mild/moderate/severe stenosis were 99/139/748, respectively.

### Association of NLR and stenosis distribution

Baseline clinical data and laboratory measurements of patients with non-stenosis/intracranial stenosis/extracranial stenosis/combined intracranial and extracranial stenosis were shown in Table 1.

**Table 1:** Comparison of factors among the patients with different distribution of atherosclerotic stenosis.

	**All (*n* = 1,129)**	**Non-stenosis (*n* = 143)**	**Intracranial stenosis alone (*n* = 396)**	**Extracranial stenosis alone (*n* = 222)**	**Combined intra/extracranial stenosis (*n* = 368)**	***p***
Male, *n* (%)	805 (71.3)	92 (64.3)	255 (64.4)	180 (81.1)	278 (75.5)	**<0.001**
Age, median (IQR)	62 (55, 68)	59 (54, 65)	59 (54, 66)	64 (58, 70)	63 (57, 70)	**<0.001**
**Medical history**
Hypertension, *n* (%)	778 (68.9)	77 (53.8)	270 (68.2)	66 (29.7)	275 (74.7)	**<0.001**
Diabetes, *n* (%)	335 (29.7)	19 (13.3)	108 (27.3)	156 (70.3)	142 (38.6)	**<0.001**
Stroke, *n* (%)	271 (24.0)	26 (18.2)	96 (24.2)	66 (29.7)	92 (25.0)	0.36
Smoking, *n* (%)	363 (32.2)	45 (31.5)	115 (29.0)	156 (70.3)	130 (35.3)	0.32
WBC, median (IQR), 10^9^/L	6.36 (5.17, 7.68)	6.09 (4.98, 7.64)	6.28 (5.11, 7.59)	6.16 (4.98, 7.40)	6.62 (5.39, 7.83)	**0.03**
Platelet, median (IQR), 10^9^/L	199.00 (165.00, 237.00)	201.00 (164.50, 235.00)	197.50 (164.75, 234.25)	188.00 (158.25, 224.00)	208.00 (173.00, 248.00)	**0.002**
Neutrophil, median (IQR), 10^9^/L	4.00 (3.00, 5.02)	3.57 (2.80, 4.70)	3.95 (3.00, 5.10)	3.91 (3.00, 4.92)	4.26 (3.11, 5.13)	**0.002**
Lymphocyte, median (IQR), 10^9^/L	1.60 (1.30, 2.00)	1.73 (1.40, 2.32)	1.57 (1.20, 2.00)	1.50 (1.21, 1.81)	1.60 (1.30, 1.96)	**<0.001**
Monocyte, median (IQR), 10^9^/L	0.50 (0.40, 0.60)	0.50 (0.40, 0.60)	0.49 (0.39, 0.60)	0.50 (0.40, 0.61)	0.50 (0.40, 0.60)	**0.04**
TC, median (IQR), mmol/L	4.01 (3.33, 4.79)	4.09 (3.47, 4.88)	3.96 (3.26, 4.80)	3.90 (3.31, 4.59)	4.08 (3.38, 4.78)	0.32
TG, median (IQR), mmol/L	1.40 (0.99, 1.89)	1.37 (0.95, 1.94)	1.42 (0.98, 1.89)	1.33 (0.98, 1.73)	1.46 (1.08, 1.96)	**0.02**
HDL, median (IQR), mmol/L	0.99 (0.85, 1.16)	1.04 (0.92, 1.22)	0.97 (0.83, 1.15)	1.04 (0.86, 1.17)	0.96 (0.84, 1.13)	**0.001**
LDL, median (IQR), mmol/L	2.43 (1.89, 3.08)	2.47 (1.94, 3.00)	2.34 (1.85, 3.10)	2.38 (1.88, 3.05)	2.48 (1.91, 3.08)	0.52
Lpa, median (IQR), mg/L	130.80 (61.20, 286.80)	109.30 (57.65, 229.15)	129.55 (57.70, 261.47)	121.45 (62.50, 319.60)	145.55 (63.17, 331.22)	0.052
MHR, median (IQR)	0.50 (0.37, 0.65)	0.48 (0.34, 0.62)	0.49 (0.36, 0.63)	0.48 (0.37, 0.64)	0.52 (0.40, 0.68)	**0.010**
NLR, median (IQR)	2.39 (1.84, 3.42)	2.08 (1.57, 2.49)	2.39 (1.90, 3.33)	2.60 (1.90, 3.65)	2.50 (1.90, 3.68)	**<0.001**

WBC, white blood cell; TC, total cholesterol; TG, triglyceride; HDL, high-density lipoprotein; LDL, low-density lipoprotein; Lpa, Lipoprotein(a); MHR, monocyte to HDL ratio; NLR, neutrophil-to-lymphocyte ratio; IQR, interquartile range; *p* for comparisons between intracranial stenosis/extracranial stenosis/combined intracranial and extracranial stenosis. The bold values indicate the significant values (*p* < 0.05).

**Figure 2:**
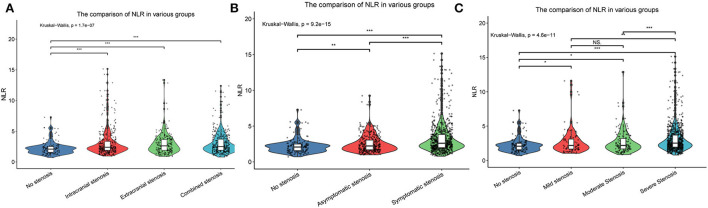
The violin plots demonstrating the distribution of the NLR levels among patients in different groups: **(A)** Without stenosis, intracranial stenosis, extracranial stenosis, and combined intracranial and extracranial stenosis; **(B)** Asymptomatic stenosis and symptomatic stenosis; **(C)** Mild stenosis, moderate stenosis, and severe stenosis. NLR, neutrophil-to-lymphocyte ratio. *p < 0.05, **p < 0.01, ***p < 0.001. NS: not significant.

As presented in Table 2, univariate and multivariate logistic regression revealed that factors associated with intracranial atherosclerosis alone were diabetes, lower HDL, and higher NLR; factors associated with extracranial atherosclerosis alone were male sex, diabetes, older age, and higher NLR; factors associated with combined intracranial and extracranial atherosclerosis were male sex, hypertension, diabetes, older age, lower HDL, higher Lpa, and higher NLR. The level of NLR in patients with different stenosis distribution was demonstrated by violin plots in Figure 2A.

**Table 2:** Univariate and multivariate logistic regression analysis of factors for the distribution of atherosclerotic stenosis.

**Variables**	**Intracranial stenosis alone**				**Extracranial stenosis alone**				**Combined intracranial and extracranial stenosis**			
	**Univariate analysis**		**Multivariate analysis**		**Univariate analysis**		**Multivariate analysis**		**Univariate analysis**		**Multivariate analysis**	
	**OR (95%CI)**	***p***	**OR (95%CI)**	***p***	**OR (95%CI)**	***p***	**OR (95%CI)**	***p***	**OR (95%CI)**	***p***	**OR (95%CI)**	***p***
Sex, M	1.00 (0.67–1.49)	0.999	–	–	2.38 (1.47–3.84)	<0.001	**2.45 (1.41–4.27)**	**0.002**	1.71 (1.13–2.60)	0.011	**1.86 (1.10–3.14)**	**0.020**
Age	1.01 (0.99–1.03)	0.467	–	–	1.07 (1.05–1.11)	<0.001	**1.07 (1.03–1.10)**	**<0.001**	1.06 (1.04–1.09)	<0.001	**1.07 (1.04–1.10)**	**<0.001**
Hypertension	1.84 (1.24–2.72)	0.002	1.43 (0.94–2.17)	0.094	2.03 (1.31–3.14)	0.002	1.43 (0.87–2.34)	0.115	2.53 (1.69–3.80)	<0.001	**1.99 (1.24–3.18)**	**0.004**
Diabetes	2.45 (1.44–4.16)	0.001	**2.23 (1.28–3.87)**	**0.005**	2.76 (1.57–4.84)	<0.001	**2.44 (1.30–4.60)**	**0.006**	4.10 (2.42–6.94)	<0.001	**3.62 (2.05–6.41)**	**<0.001**
Stroke	1.44 (0.34–2.33)	0.139	–	–	1.55 (0.592–2.62)	0.097	–	–	1.50 (0.92–2.44)	0.102	–	–
Smoking	0.89 (0.59–1.35)	0.586	–	–	1.07 (0.68–1.67)	0.778	–	–	1.19 (0.79–1.80)	0.41	–	–
WBC	1.09 (0.99–1.19)	0.081	–	–	1.04 (0.93–1.17)	0.454	–	–	1.16 (1.04–1.29)	0.008	0.99 (0.84–1.17)	0.912
Platelet	1.00 (1.00–1.00)	0.606	–	–	1.00 (0.99–1.00)	0.343	–	–	1.00 (1.00–1.01)	0.048	1.00 (1.00–1.01)	0.064
Neutrophil	1.22 (1.08–1.38)	0.001	–	–	1.19 (1.03–1.38)	0.017	–	–	1.32 (1.15–1.52)	<0.001	–	–
Lymphocyte	0.48 (0.34–0.69)	<0.001	–	–	0.45 (0.30–0.66)	<0.001	–	–	0.55 (0.39–2.60)	0.001	–	–
Monocyte	0.98 (0.35–0.69)	0.971	–	–	1.62 (0.49–5.28)	0.430	–	–	2.82 (0.90–2.44)	0.076	–	–
TC	0.92 (0.78–1.18)	0.624	–	–	0.84 (0.69–1.03)	0.090	–	–	0.98 (0.83–1.16)	0.802	–	–
TG	1.00 (0.87–1.15)	0.955	–	–	0.84 (0.67–1.05)	0.120	–	–	1.02 (0.87–1.19)	0.843	–	–
HDL	0.36 (0.18–0.73)	0.005	**0.36 (0.17–0.76)**	**0.008**	0.37 (0.15–0.89)	0.027	0.43 (0.15–1.27)	0.128	0.22 (0.10–0.48)	<0.001	**0.15 (0.04–0.53)**	**0.003**
LDL	0.95 (0.76–1.18)	0.624	–	–	0.92 (0.72–1.17)	0.449	–	–	1.05 (0.85–1.30)	0.643	–	–
Lpa	1.00 (1.00–1.00)	0.253	–	–	1.00 (1.00–1.00)	0.032	1.00 (1.00–1.00)	0.096	1.00 (1.00–1.00)	0.016	**1.00 (1.00–1.00)**	**0.017**
MHR	1.79 (0.80–3.99)	0.157	–	–	1.95 (0.75–5.04)	0.168	–	–	3.94 (1.61–9.62)	0.003	0.51 (0.11–2.27)	0.375
NLR	1.51 (1.25–1.82)	<0.001	**1.54 (1.27–1.85)**	**<0.001**	1.60 (1.29–1.98)	<0.001	**1.56 (1.25–1.96)**	**<0.001**	1.64 (1.34–2.00)	<0.001	**1.61 (1.28–2.03)**	**<0.001**

WBC, white blood cell; TC, total cholesterol; TG, triglyceride; HDL, high-density lipoprotein; LDL, low-density lipoprotein; Lpa, Lipoprotein(a); MHR, monocyte to HDL ratio; NLR, neutrophil-to-lymphocyte ratio; OR, odds ratio; 95% CI, 95% confidence interval; multivariable analysis: adjusted for factors with *p* < 0.05 in univariate analysis, neutrophil and lymphocyte were not included due to multicollinearity. The bold values indicate the significant values (*p* < 0.05).

**Figure 3:**
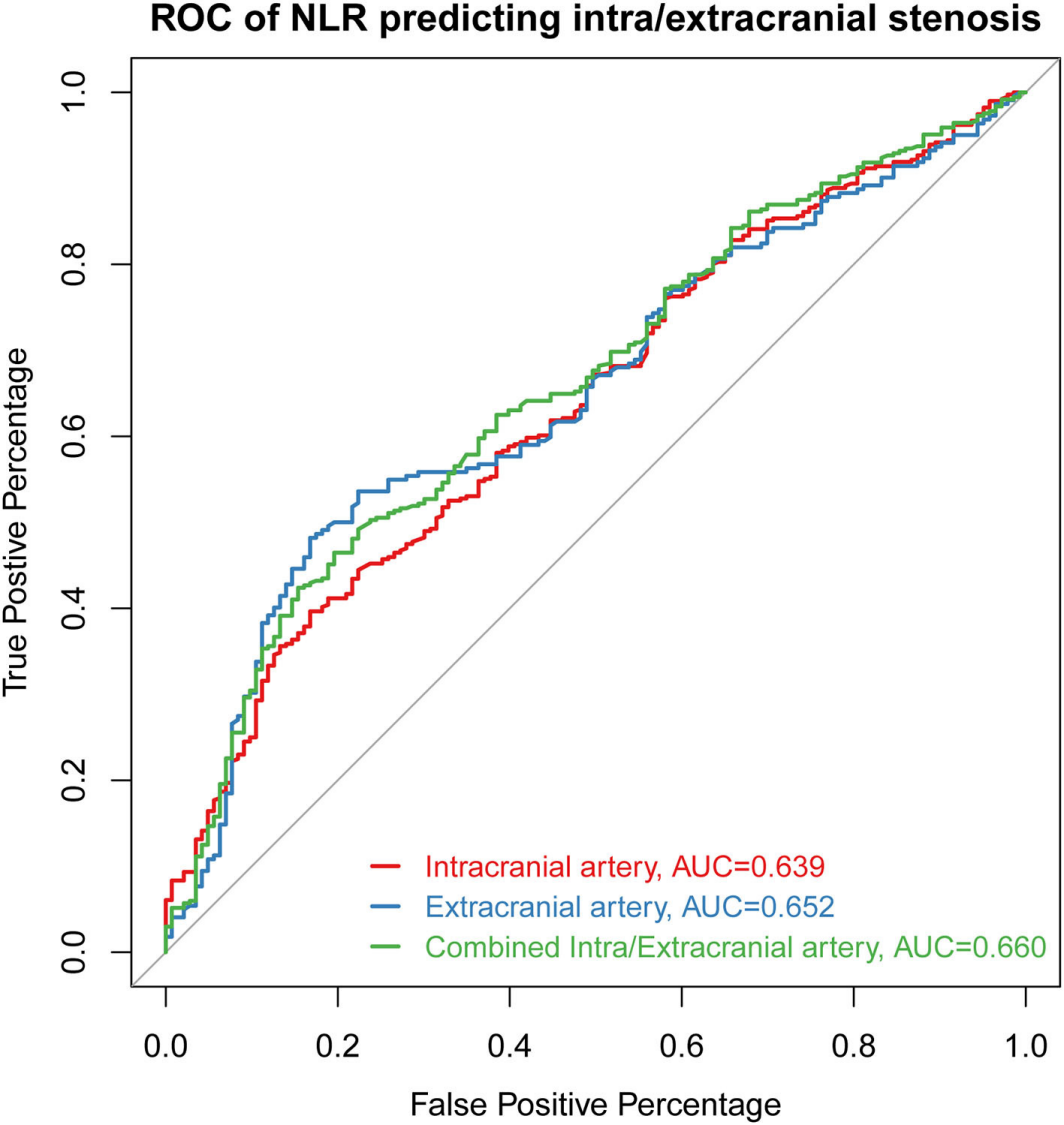
The ROC curve of NLR for predicting intracranial stenosis, extracranial stenosis, and combined intracranial and extracranial stenosis. ROC, receiver operating characteristic curve; AUC, area under the curve; NLR, neutrophil-to-lymphocyte ratio.

Receiver operating characteristic curves presenting the value of NLR in predicting stenosis distribution were shown in Figure 3. The AUC of NLR for predicting intracranial stenosis, extracranial stenosis, and combined intracranial and extracranial stenosis were 0.639 (95% CI 0.588–0.690, *p* < 0.001), 0.652 (95% CI 0.595–0.709, *p* < 0.001), and 0.660 (95% CI 0.609–0.711, *p* < 0.001). The cut off values were 2.688 with 39.6% sensitivity and 83.3% specificity, 2.689 with 48.2% sensitivity and 83.2% specificity, 2.764 with 42.4% sensitivity, and 84.6% specificity, respectively.

### Association of NLR and asymptomatic/symptomatic stenosis

**Figure 4:**
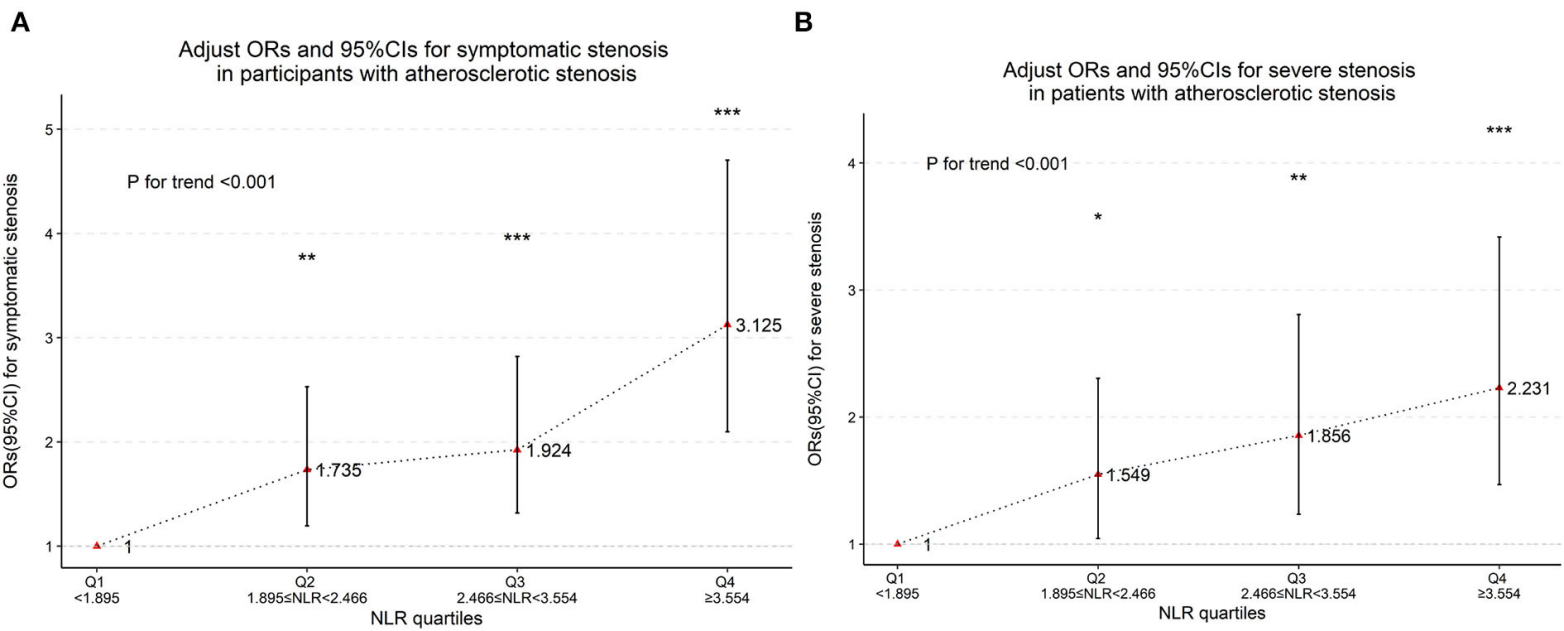
Multinomial-adjusted odds ratio (Ors) and 95% confidence interval (95%Cis) for symptomatic stenosis **(A)** and severe stenosis **(B)** according to neutrophil-to-lymphocyte ratio (NLR) quartiles. Ors were adjusted for “sex,” “age,” “hypertension,” “diabetes,” “stroke,” and “smoking.” NLR was significantly associated with severe stenosis and symptomatic stenosis in the second, third and fourth quartiles (the first NLR quartile as reference). *p < 0.05, **p < 0.01, ***p < 0.001, *p* for trend <0.001.

Baseline clinical data and laboratory measurements of the non-stenosis/asymptomatic stenosis/symptomatic stenosis patients were shown in Table 3. As presented in Table 4, compared with the asymptomatic stenosis group, the symptomatic stenosis patients presented a greater male proportion, smoker proportion, neutrophil level, monocyte level, MHR, and NLR level, while the symptomatic group had lower hypertension proportion, age, and white blood cell (WBC) level. After multivariate analysis, smoking, younger age, and higher NLR were independently associated with symptomatic stenosis. The violin plots demonstrated the distribution of NLR in non-stenosis/asymptomatic stenosis/symptomatic stenosis groups in Figure 2B. The covariates-adjusted result for symptomatic stenosis according to NLR quartiles was shown in Figure 4A. Compared with the first quartile, the second (OR = 1.74, 95% CI 1.19–2.53), third (OR =1.92, 95% CI 1.32–2.82), and fourth (OR = 3.13, 95% CI 2.10–4.70) quartiles were independent risk factors for symptomatic stenosis (*p* for trend < 0.001).

**Table 3:** Comparison of factors among the patients in the non-stenosis group, asymptomatic stenosis group and symptomatic stenosis group.

	**Non-stenosis (*n* = 143)**	**Asymptomatic stenosis (*n* = 318)**	**Symptomatic stenosis (*n* = 668)**	***p***
Male, *n* (%)	92 (64.3%)	209 (65.7%)	504 (75.4%)	**0.001**
Age, median (IQR)	59 (54, 65)	64 (57, 71)	61 (55, 68)	**<0.001**
**Medical history**				
Hypertension, *n* (%)	77 (53.8)	241 (75.8)	460 (68.9)	**<0.001**
Diabetes, *n* (%)	19 (13.3)	104 (32.7)	212 (31.7)	**<0.001**
Stroke, *n* (%)	26 (18.2)	85 (26.7)	160 (24.0)	0.139
Smoking, *n* (%)	45 (31.5)	75 (23.6)	243 (36.4)	**<0.001**
WBC, median (IQR), 10^9^/L	6.09 (4.98, 7.64)	5.94 (5.00, 7.10)	6.67 (5.30, 8.00)	**<0.001**
Platelet, median (IQR), 10^9^/L	201.00 (164.50, 235.00)	196.00 (168.00, 233.00)	201.00 (165.00, 240.00)	0.485
Neutrophil, median (IQR), 10^9^/L	3.57 (2.80, 4.70)	3.60 (2.89, 4.60)	4.30 (3.16, 5.38)	**<0.001**
Lymphocyte, median (IQR), 10^9^/L	1.73 (1.40, 2.32)	1.62 (1.30, 1.97)	1.56 (1.20, 1.94)	**<0.001**
Monocyte, median (IQR), 10^9^/L	0.50 (0.40, 0.60)	0.50 (0.38, 0.60)	0.50 (0.40, 0.61)	**0.048**
TC, median (IQR), mmol/L	4.09 (3.47, 4.88)	3.88 (3.22, 4.72)	4.04 (3.37, 4.81)	0.136
TG, median (IQR), mmol/L	1.37 (0.95, 1.94)	1.48 (1.01, 1.93)	1.38 (0.99, 1.87)	0.362
HDL, median (IQR), mmol/L	1.04 (0.92, 1.22)	1.00 (0.86, 1.14)	0.98 (0.83, 1.16)	**0.002**
LDL, median (IQR), mmol/L	2.47 (1.94, 3.00)	2.32 (1.77, 2.96)	2.44 (1.92, 3.14)	0.062
Lpa, median (IQR), mg/L	109.30 (57.65, 229.15)	142.30 (59.78, 331.88)	131.40 (62.40, 286.05)	0.096
MHR, median (IQR)	0.48 (0.34, 0.62)	0.48 (0.36, 0.62)	0.51 (0.38, 0.68)	**0.014**
NLR, median (IQR)	2.08 (1.57, 2.49)	2.22 (1.70, 3.04)	2.60 (2.00, 3.83)	**<0.001**

WBC, white blood cell; TC, total cholesterol; TG, triglyceride; HDL, high-density lipoprotein; LDL, low-density lipoprotein; Lpa, Lipoprotein(a); MHR, monocyte to HDL ratio; NLR, neutrophil-to-lymphocyte ratio; *p* for comparisons between asymptomatic and symptomatic stenosis. The bold values indicate the significant values (*p* < 0.05).

**Table 4:** Univariate and multivariate logistic regression of factors for the asymptomatic and symptomatic stenosis.

**Variables**	**Univariate analysis**			**Multivariate analysis**		
	**OR**	**95% CI**	***p***	**OR**	**95% CI**	***p***
Sex, M	1.60	1.20–2.14	**0.001**	1.25	0.89–1.75	0.191
Age	0.97	0.96–0.99	**<0.001**	**0.97**	**0.96–0.99**	**0.001**
Hypertension	0.71	0.52–0.96	**0.025**	0.78	0.57–1.07	0.127
Diabetes	0.96	0.72–1.27	0.761	–	–	–
Stroke	0.86	0.64–1.17	0.346	–	–	–
Smoking	1.85	1.37–2.51	**<0.001**	**1.67**	**1.19–2.33**	**0.003**
WBC	1.19	1.10–1.27	**<0.001**	1.06	0.96–1.18	0.248
Platelet	1.00	1.00–1.00	0.198	–	–	–
Neutrophil	1.27	1.16–1.38	**<0.001**	–	–	–
Lymphocyte	0.80	0.62–1.03	0.087	–	–	–
Monocyte	2.56	1.19–5.50	**0.016**	0.47	0.09–2.38	0.360
TC	1.07	0.95–1.20	0.290	–	–	–
TG	0.93	0.83–1.04	0.200	–	–	–
HDL	0.88	0.51–1.52	0.655	–	–	–
LDL	1.16	1.00–1.35	0.051	–	–	–
Lpa	0.95	0.84–1.08	0.446	–	–	–
MHR	2.06	1.17–3.62	**0.012**	1.56	0.52–4.64	0.424
NLR	1.33	1.20–1.48	**<0.001**	**1.32**	**1.17–1.49**	**<0.001**

WBC, white blood cell; TC, total cholesterol; TG, triglyceride; HDL, high-density lipoprotein; LDL, low-density lipoprotein; Lpa, Lipoprotein(a); MHR, monocyte to HDL ratio; NLR, neutrophil-to-lymphocyte ratio; OR, odds ratio; 95% CI, 95% confidence interval; multivariable analysis: adjusted for factors with *p* < 0.05 in univariate analysis, neutrophil and lymphocyte were not included due to multicollinearity. The bold values indicate the significant values (*p* < 0.05).

### Association of NLR and degree of stenosis

Baseline clinical data and laboratory measurements of the patients with mild/moderate/severe stenosis were shown in Table 5. Univariate analysis indicated that neutrophil and NLR were increased with the severity of stenosis degree (Table 6). The violin plots demonstrated the distribution of NLR with stenosis degree in Figure 2C. The covariates-adjusted result for severe stenosis according to NLR quartiles was shown in Figure 4B. Compared with the first quartile, the second (OR = 1.55, 95% CI 1.05–2.31), third (OR =1.86, 95% CI 1.24–2.81), and fourth (OR = 2.23, 95% CI 1.47–3.42) quartiles were independent risk factors for severe stenosis (*p* for trend < 0.001).

**Table 5:** Comparison of factors among patients in mild stenosis group, moderate stenosis group, and severe stenosis group.

	**All (*n* = 986)**	**Mild stenosis (*n* = 99)**	**Moderate stenosis (*n* = 139)**	**Severe stenosis (*n* = 748)**	***p***
Male, *n* (%)	713 (72.3)	68 (68.7)	95 (68.3)	550 (73.5)	0.32
Age, median (IQR)	62.33 (8.61)	63.87 (7.91)	61.86 (8.13)	62.22 (8.77)	0.16
**Medical history**
Hypertension, *n* (%)	701 (71.1)	66 (66.7)	105 (75.5)	530 (70.9)	0.32
Diabetes, *n* (%)	316 (32.0)	27 (27.3)	45 (32.4)	244 (32.6)	0.56
Stroke, *n* (%)	245 (24.8)	26 (26.3)	35 (25.2)	184 (24.6)	0.93
Smoking, *n* (%)	318 (32.3)	32 (32.3)	48 (34.5)	238 (31.8)	0.82
WBC, median (IQR), 10^9^/L	6.39 (5.20, 7.68)	5.94 (4.76, 7.12)	6.00 (5.10, 7.45)	6.54 (5.28, 7.80)	**0.007**
Platelet, median (IQR), 10^9^/L	199.00 (165.00, 237.00)	194.00 (158.50, 234.00)	215.00 (175.50, 250.50)	198.00 (164.00, 236.25)	**0.018**
Neutrophil, median (IQR), 10^9^/L	4.03 (3.02, 5.10)	3.56 (2.80, 4.62)	3.72 (2.99, 4.80)	4.20 (3.10, 5.22)	**<0.001**
Lymphocyte, median (IQR), 10^9^/L	1.57 (1.25, 1.96)	1.51 (1.20, 2.08)	1.66 (1.35, 2.02)	1.56 (1.23, 1.91)	0.15
Monocyte, median (IQR), 10^9^/L	0.50 (0.40, 0.60)	0.50 (0.38, 0.60)	0.50 (0.40, 0.61)	0.50 (0.40, 0.60)	0.61
TC, median (IQR), mmol/L	3.99 (3.31, 4.77)	4.20 (3.45, 4.73)	4.03 (3.38, 4.85)	3.95 (3.29, 4.74)	0.46
TG, median (IQR), mmol/L	1.40 (1.00, 1.88)	1.47 (0.98, 1.90)	1.37 (1.02, 1.88)	1.40 (1.00, 1.88)	0.96
HDL, median (IQR), mmol/L	0.98 (0.84, 1.15)	1.00 (0.84, 1.12)	0.97 (0.82, 1.16)	0.98 (0.84, 1.15)	0.86
LDL, median (IQR), mmol/L	2.41 (1.88, 3.08)	2.52 (1.99, 3.17)	2.41 (1.88, 3.26)	2.40 (1.85, 3.05)	0.57
Lpa, median (IQR), mg/L	135.35 (61.32, 303.20)	128.20 (55.95, 270.10)	136.20 (62.50, 278.30)	135.85 (61.58, 313.25)	0.39
MHR, median (IQR)	0.50 (0.37, 0.65)	0.50 (0.36, 0.70)	0.52 (0.36, 0.66)	0.50 (0.37, 0.65)	0.95
NLR, median (IQR)	2.47 (1.90, 3.55)	2.19 (1.67, 3.11)	2.19 (1.70, 3.19)	2.53 (1.96, 3.69)	**<0.001**

WBC, white blood cell; TC, total cholesterol; TG, triglyceride; HDL, high-density lipoprotein; LDL, low-density lipoprotein; Lpa, Lipoprotein(a); MHR, monocyte to HDL ratio; NLR, neutrophil-to-lymphocyte ratio; *p* for comparisons between mild/moderate/severe stenosis. The bold values indicate the significant values (*p* < 0.05).

**Table 6:** Univariate ordinal logistic regression of factors for the mild stenosis group, moderate stenosis group, and severe stenosis group.

**Variables**	**Univariate analysis**		
	**OR**	**95% CI**	***p***
Sex, M	1.27	0.93–1.74	0.14
Age	0.99	0.98–1.01	0.33
Hypertension	0.99	0.72–1.36	0.93
Diabetes	1.13	0.83–1.55	0.43
Stroke	0.94	0.68–1.32	0.73
Smoking	0.93	0.68–1.27	0.64
WBC	1.07	0.99–1.15	0.07
Platelet	1.00	1.00–1.00	0.30
Neutrophil	1.21	1.10–1.33	**<0.001**
Lymphocyte	0.84	0.64–1.10	0.20
Monocyte	1.68	0.75–3.80	0.21
TC	0.96	0.84–1.10	0.57
TG	0.97	0.86–1.10	0.64
HDL	1.15	0.63–2.08	0.66
LDL	0.98	0.84–1.15	0.79
Lpa	1.00	1.00–1.00	0.10
MHR	1.04	0.59–1.82	0.90
NLR	1.16	1.05–1.27	**0.003**

WBC, white blood cell; TC, total cholesterol; TG, triglyceride; HDL, high-density lipoprotein; LDL, low-density lipoprotein; Lpa, Lipoprotein(a); MHR, monocyte to HDL ratio; NLR, neutrophil-to-lymphocyte ratio; OR, odds ratio, 95%CI, 95% confidence interval. *P* = 0.396 > 0.05 for ordered logistic parallel line test. The bold value indicates the significant values (*p* < 0.05).

## Discussion

In the current study, the “gold standard” of cerebrovascular DSA examination was used to diagnose intracranial and extracranial stenosis. Our results demonstrated that NLR is associated with both intracranial and extracranial atherosclerotic stenosis. Patients with symptomatic intracranial/extracranial atherosclerotic stenosis or a more severe degree of stenosis presented elevated NLR.

Neutrophil-to-lymphocyte ratio is easily obtained by dividing the absolute neutrophil count by lymphocyte count from peripheral complete blood counts and has been regarded as a simple index of the inflammatory response. Previous studies have suggested NLR as a reliable prognostic biomarker in various inflammation processes, such as acute cerebral hemorrhage (21, 22), acute coronary syndrome (23), and solid tumors (24).

Atherosclerosis is a common underlying pathology of cerebrovascular disease; it advances with age and is more severe in elder subjects (25). Atherosclerosis has been considered a cholesterol storage disease in the intimal space of arteries and correlated with plasma cholesterol and apolipoproteins levels (26). In addition, the inflammatory response is also implicated in the pathogenesis of atherosclerosis, with the accumulation of immune cells into the atherosclerotic plaque (3). Neutrophil is a proinflammatory cell that plays a pivotal role in the innate immune response including phagocytosis and cytokines release. It is involved in the initiation and progression of atherosclerosis, namely endothelial function aggravation, foam cell formation, plaque destabilization, fibrous cap weakening, and endothelial erosion (27). Neutrophil could be recruited and activated on the vascular wall, secreting granule proteins, and limiting the use of nitric oxide, thus leading to endothelial dysfunction and subsequent atherosclerosis (27). Also, neutrophil releases extracellular traps, inducing macrophage to release cytokines and activate Th-17 cells, further amplifying immune cell recruitment in atherosclerotic plaque and causing plaque destabilization and rupture (28, 29). Lymphocyte has been reported to upregulate the anti-inflammatory cytokine interleukin (IL)-10 and suppress inflammatory cytokines including tumor necrosis factor (TNF)-α and IL-6, which can play an anti-inflammatory effect (30). Doran et al. indicated that B lymphocytes had protective effects on atherosclerosis formation (31). Saigusa et al. described *T* cells as critical drivers and modifiers of the pathogenesis of atherosclerosis, Th1 cells had a pro-atherosclerotic characteristic, and Treg cells played an anti-atherogenic role (32). Therefore, NLR may actively participate in the process of atherosclerosis, and is closely related to intracranial and extracranial atherosclerotic stenosis.

In our cohort, 71.3% of the patients were male while 28.7% were female. This gender difference could be because of the influence of sex hormones on vascular inflammation-mediated atherosclerosis, as presented in previous studies (33). There were more patients with intracranial stenosis than with extracranial stenosis in our study. This is in line with the ethnic difference of stenosis distribution. Evidence has demonstrated that compared to Europe and America, intracranial arterial stenosis is more common in Asia (34). The distribution classification is because of the morphological and biological differences between intracranial and extracranial arteries. Compared with the extracranial arteries, intracranial arteries are muscle arteries and have been suggested to have greater resistance to atherosclerosis. Our study demonstrated that NLR correlated with intracranial stenosis, extracranial stenosis, and combined intracranial/extracranial stenosis. Our finding of high NLR level in intracranial stenosis is in accordance with previous studies. Chung et al. have revealed that high NLR was correlated with intracranial large-artery atherosclerosis in individuals without neurological diseases (35). Nam et al. also reported that NLR was associated with both prevalence and burdens of intracranial atherosclerosis in a healthy population (13). Moreover, Ying et al. concluded that elevated admission NLR level was an independent factor associated with stroke severity and prognosis in symptomatic intracranial atherosclerosis patients (6). The relationship between extracranial large-artery stenosis and elevated NLR level was also reported by Chung et al. in line with our results (35).

Our findings showed that patients with symptomatic stenosis presented higher NLR. Given the role of NLR in plaque destabilization, it is not surprising that patients with symptomatic stenosis presented higher NLR level (27). The association between high NLR and symptomatic internal carotid stenosis was reported by Massiot et al. (14). Ying et al. demonstrated that NLR was associated with stroke severity and prognosis after ischemic stroke in patients with symptomatic intracranial stenosis (6).

The severity of stenosis and NLR level was also discussed in our study. Patients with more severe stenosis presented higher NLR level. Jiang et al. pointed out that NLR correlated with the severity of extracranial carotid stenosis, and a cut off value of NLR at 1.89 for predicting ≥50% maximal extracranial carotid stenosis was proposed, with 78.4% sensitivity and 77.4% specificity (36).

Other cerebrovascular risk factors, such as age, sex, hypertension, diabetes, smoking, Lipoprotein(a), and low HDL, were also shown to be associated with atherosclerotic stenosis in our cohort. However, contrary to a previous study (15), MHR did not present an independent association with atherosclerotic stenosis in our cohort in multivariate analysis. This may suggest that NLR is more closely related to atherosclerotic stenosis.

This study has several limitations. First, we only selected NLR and MHR as inflammation indicators, other markers were not included due to unavailability, such as C-reaction protein and interleukin. Second, we did not include neurological severity as outcome variable, such as the modified Rankin Scale. Third, our results could not establish a causal association between NLR and intracranial/extracranial stenosis because of the cross-sectional study design.

## Conclusion

In conclusion, the present study demonstrated that NLR is an important factor associated with both intracranial and extracranial atherosclerotic stenosis. Patients with symptomatic intracranial/extracranial atherosclerotic stenosis or a more severe degree of stenosis presented elevated NLR.

## Data availability statement

The data that support the findings of this study are available from the corresponding author upon reasonable request.

## Ethics statement

The studies involving human participants were reviewed and approved by the Clinical Research Ethics Committee of Zhongnan Hospital of Wuhan University. Written informed consent for participation was not required for this study in accordance with the national legislation and the institutional requirements.

## Author contributions

YX, ZL, SR, and YL contributed to the conception and design of the study. YX, ZL, BD, LZo, LZh, RZ, HL, QC, and NA were performed data collection, statistical analysis, and table and figure preparation. All authors drafted text and proofed the final version of the manuscript.

## Funding

This work was supported by the Program of Excellent Doctoral (Postdoctoral) of Zhongnan Hospital of Wuhan University, Grant/Award Number: ZNYB2019005.

## Conflict of interest

The authors declare that the research was conducted in the absence of any commercial or financial relationships that could be construed as a potential conflict of interest.

## Publisher's note

All claims expressed in this article are solely those of the authors and do not necessarily represent those of their affiliated organizations, or those of the publisher, the editors and the reviewers. Any product that may be evaluated in this article, or claim that may be made by its manufacturer, is not guaranteed or endorsed by the publisher.
